# Periodized Nutrition for Athletes

**DOI:** 10.1007/s40279-017-0694-2

**Published:** 2017-03-22

**Authors:** Asker E Jeukendrup

**Affiliations:** School of Sport, Exercise and Health Sciences, Loughborough University, Loughborough, Leicestershire LE11 3TU UK

## Abstract

It is becoming increasingly clear that adaptations, initiated by exercise, can be amplified or reduced by nutrition. Various methods have been discussed to optimize training adaptations and some of these methods have been subject to extensive study. To date, most methods have focused on skeletal muscle, but it is important to note that training effects also include adaptations in other tissues (e.g., brain, vasculature), improvements in the absorptive capacity of the intestine, increases in tolerance to dehydration, and other effects that have received less attention in the literature. The purpose of this review is to define the concept of periodized nutrition (also referred to as nutritional training) and summarize the wide variety of methods available to athletes. The reader is referred to several other recent review articles that have discussed aspects of periodized nutrition in much more detail with primarily a focus on adaptations in the muscle. The purpose of this review is not to discuss the literature in great detail but to clearly define the concept and to give a complete overview of the methods available, with an emphasis on adaptations that are not in the muscle. Whilst there is good evidence for some methods, other proposed methods are mere theories that remain to be tested. ‘Periodized nutrition’ refers to the strategic combined use of exercise training and nutrition, or nutrition only, with the overall aim to obtain adaptations that support exercise performance. The term nutritional training is sometimes used to describe the same methods and these terms can be used interchangeably. In this review, an overview is given of some of the most common methods of periodized nutrition including ‘training low’ and ‘training high’, and training with low- and high-carbohydrate availability, respectively. ‘Training low’ in particular has received considerable attention and several variations of ‘train low’ have been proposed. ‘Training-low’ studies have generally shown beneficial effects in terms of signaling and transcription, but to date, few studies have been able to show any effects on performance. In addition to ‘train low’ and ‘train high’, methods have been developed to ‘train the gut’, train hypohydrated (to reduce the negative effects of dehydration), and train with various supplements that may increase the training adaptations longer term. Which of these methods should be used depends on the specific goals of the individual and there is no method (or diet) that will address all needs of an individual in all situations. Therefore, appropriate practical application lies in the optimal combination of different nutritional training methods. Some of these methods have already found their way into training practices of athletes, even though evidence for their efficacy is sometimes scarce at best. Many pragmatic questions remain unanswered and another goal of this review is to identify some of the remaining questions that may have great practical relevance and should be the focus of future research.

## Introduction

The adaptive response to exercise training is determined by a combination of factors: the duration, the intensity, and the type of exercise as well as the frequency of training, but also by the quality and quantity of nutrition in the pre- and post-exercise periods. It is becoming increasingly clear that adaptations, initiated by exercise, can be amplified or dampened by nutrition. For example, it is well established that in the absence of protein feeding post-exercise, net protein synthesis is low and the muscle may actually be in negative protein balance. There is also evidence that lowering carbohydrate availability can promote specific adaptations in the muscle. In contrast, high-dose antioxidant supplementation has the potential to reduce training adaptations [1–3]. Research has mostly focused on adaptations in skeletal muscle. Critically, there are many adaptations in other organs that are influenced by nutritional intake and that are important to sports performance. Such changes and their relevance for athletes are often overlooked or have received significantly less attention. Examples include, but are not limited to, the vasculature, the brain, and the intestine. For example, there is evidence of the upregulation of carbohydrate transporters in the intestine in response to carbohydrate feeding and there are alterations in gut micro flora in response to changes in diet. Such changes could alter the delivery of nutrients and potentially affect performance.

There are thus numerous interactions between nutrition and exercise and numerous effects of nutrition per se that ultimately determine long-term exercise performance outcomes. From a practical point of view, it is important to have an understanding of these interactions to optimize specific adaptations that one might be interested in. There are numerous reviews that have discussed aspects of this. For example, several reviews have discussed the potential benefits of training with low-carbohydrate availability [4–6], some have discussed high-carbohydrate availability or both [7, 8], others have discussed the potential impact of antioxidants on mitochondrial biogenesis [9] or other modulators of training adaptation [10]. The purpose of this review is two fold: to clearly define the concept of periodized nutrition, and to provide a holistic overview of the methods that could fall under the umbrella term ‘periodized nutrition’.

## Historical Perspective

The links between diet and exercise have long been recognized. In the late 1800s, the term training was used to describe a regime that included diet as well as exercise, not just exercise. Training was and is still often defined as “the action of undertaking a course of exercise and diet in preparation for a sporting event”. At one point in history, nutrition was such an important part of athletes’ preparation that the definition of training was more related to diet than the actual physical preparation itself. Below are some excerpts from a book on training by Montague Shearman in 1887 [11].

“To the athlete of early times, the essential part and chief characteristic of training was not the taking of proper preparatory exercise, but the sudden and violent change of diet”. “Going into training was taken to mean the commencement of a peculiar diet of half-cooked beefsteaks and dry bread and the reduction of the daily drink to a minimum, and not to imply the beginning of the proper training or cultivation of the muscles required for a race.”

Although these practices themselves may not have stood the test of time and may not be supported by scientific evidence, it is clear that also in those early days, a clear link was assumed between diet and exercise performance. Although these effects aim at short-term performance benefits, more recently, studies have focused on longer term effects. It has been suggested that by careful planning and integration of nutrition and training, the longer term training adaptations might be improved. The terms ‘periodized nutrition’ and ‘nutritional training’ are sometimes used to refer to such strategies.

## What is Periodized Nutrition?

It is important to define the terms ‘periodized nutrition’ and ‘nutritional training’. The words ‘training’ and ‘periodized’ by definition refer to a structured and planned process. In reality, there is often little planning when it comes to nutrition and limited integration of training and nutritional practices. What athletes consume post-exercise may depend on the training, but careful planning ahead of training, with long-term goals in mind, is still relatively uncommon. Clear guidelines are still lacking as this developing field of research is only in its infancy. Most nutritional recommendations for athletes aim to promote acute recovery after exercise without acknowledging the specific goal of the exercise and often without taking into account the severity and type of exercise or the longer term goals.

In this review, I provide an overview of potential strategies aimed at enhancing specific adaptations that could help athletic performance, and I define the term ‘periodized nutrition’. In the literature, the term ‘periodized nutrition’ is sometimes used, but a clear definition and a common interpretation is lacking. The term periodization in the context of exercise training refers to a long-term progressive approach designed to improve athletic performance by systematically varying training throughout the year. The term nutrition periodization is typically used to describe changes in nutritional intake in response to certain periods of training [12–14]. For example, during certain periods of training there is a focus on weight management and lower energy intake, whereas during other periods there is a focus on recovery and performance and higher carbohydrate intake. Mujika et al. [14] concluded that “Nutrition should be periodized and adapted to support changing individual goals, training levels, and requirements throughout a season and/or training cycle”. Hawley and Burke [4] discussed the importance of a long term periodized training-nutrition program as a way to enhance performance. The authors stated “… it seems prudent to suggest that competitive athletes may wish to manipulate carbohydrate availability before, during, or after selected training sessions that form part of a long-term periodized training-nutrition plan to promote metabolic training adaptations that should, in theory, promote endurance-based performances”. In this statement, there is a strong focus on carbohydrate availability as a driver of training effects, and the training effects are mostly in the muscle and metabolic in nature. For example, training the extension of the stomach wall as discussed in Jeukendrup [15] would not be included in this definition of periodized nutrition.

Therefore, I propose the following definition: periodized nutrition refers to the planned, purposeful, and strategic use of specific nutritional interventions to enhance the adaptations targeted by individual exercise sessions or periodic training plans, or to obtain other effects that will enhance performance longer term.

The definition of periodized nutrition (or nutritional training) introduced above includes all methods that use nutrition (in the presence or absence of training) to improve long-term performance. These methods include manipulations of nutrient availability before, during, and after training, but could also include practices that prepare other organs for competition through nutritional manipulation (e.g., improving stomach comfort by regularly drinking large volumes [15]). The definition of nutritional training is not restricted to adaptations of the muscle (and could relate to adaptations in all organs), but will always have long-term performance improvements as the main goal.

## Nutritional Training: Specific Goals Require Specific Methods

The terms periodized nutrition and nutritional training can be used interchangeably and the selection of nutritional training methods is highly specific to the goals. For example, if the goal is to develop fat metabolism specifically, there may be a role of training with low-carbohydrate availability that will achieve these specific adaptations. However, to achieve adaptations of the gastrointestinal (GI) absorptive capacity [15] for carbohydrates, an increased carbohydrate intake would be recommended. There may be a role for both of these seemingly contrasting methods in the training approach of an athlete. In the future, we are likely to see more planning of nutrition as part of the training plan of athletes. Specific workouts will be accompanied by specific nutritional goals. Nutrition can be planned as much as training can be planned and can be made more purposeful. This will also allow inter-individual differences in both physiology and goals to be taken into account.

Different nutritional training methods can be used to achieve specific goals (see Table 1). It is beyond the scope of this review to discuss all methods in great detail and several methods have been discussed at length in various excellent recently published reviews [4, 5, 7–10]. I refer to these reviews in the relevant sections, rather than discussing the same studies in detail. In this review, I only summarize the different nutritional training tools that have been studied, and explain briefly the underlying principles and potential benefits. Periodized nutrition does not refer to long-term diet composition or any form of dieting, unless this diet is strategically altered to accommodate specific needs during specific periods. In Table 1, an overview of some of the available nutritional training methods is provided. This list may not be exhaustive but it represents the most important variations that have received attention from researchers where there is at least some supporting evidence in the scientific literature.

**Table 1:** Nutritional training methods: while some methods have more supporting evidence than others, these are the potential nutritional training tools that athletes and coaches can use to periodize the athlete’s nutrition

Train low	Training twice a day	Limited or no carbohydrate intake between the two sessions. The first training will lower muscle glycogen so that the second training is performed in a low-glycogen state. This may increase the expression of relevant genes
	Training fasted	Training is performed after an overnight fast. Muscle glycogen may be normal or even high but liver glycogen is low
	Training with low exogenous carbohydrate availability	No or very little carbohydrate is ingested during prolonged exercise. This may exaggerate the stress response
	Low-carbohydrate availability during recovery	No or very little carbohydrate is ingested post-exercise. This may prolong the stress response
	Sleep low	Train late in the day and go to bed with carbohydrate intake restricted. Essentially the same idea as low-carbohydrate availability after training but the period post-exercise is extended. Muscle and liver glycogen will be low for several hours during sleep
	Low-carbohydrate high-fat/ketogenic diets	Long-term low-carbohydrate stores
Train high	Training with high muscle and liver glycogen	Carbohydrate intake is high before training when glycogen is important and there is a focus on glycogen restoration post-exercise
	Training with a high-carbohydrate diet	Carbohydrate intake is high on a daily basis independent of training, but may be especially high around training (during and after)
Training the gut	Training of stomach comfort	Increasing volume of intake with or without exercise
	Training gastric emptying	Repeated use of meals to increase/improve gastric emptying of fluids or nutrients (carbohydrate) and reduce stomach discomfort
	Training absorption	Increasing daily carbohydrate intake and/or intake during exercise to improve absorptive capacity of the gut and reduce intestinal discomfort
	Training race nutrition	Training all aspects of a nutrition strategy as on race day
Training dehydrated	Training in a dehydrated state	Training with limited/no fluid intake to allow dehydration
Improving training adaptations with supplements	Supplements	Supplements that may allow more training to be performed (see Table 2)
		Supplements that may initiate or increase protein synthesis and/or increase myofibrillar protein synthesis (see Table 2)
		Supplements with the potential to increase mitochondrial biogenesis (see Table 2)

### Training Low

Training low is a general term to describe training with low-carbohydrate availability. This low-carbohydrate availability could be low muscle glycogen, low liver glycogen, low-carbohydrate intake during or after exercise, or combinations thereof. The rationale for reducing carbohydrate availability is derived from early studies that observed links between carbohydrate availability (muscle glycogen) and gene expression [16] because it is generally believed that training adaptations are the result of accumulated small changes in protein synthesis that result in an altered phenotype and improved performance. For this protein synthesis to occur, it is important that there is a stress signal, transcription, and translation, that messenger RNA remains stable, and that sufficient amino acids are available for protein synthesis. Many of these factors are influenced by nutrition. For example, the metabolic changes that occur as a result of muscle contraction, including a rise in AMP-activated protein kinase (AMPK), are important factors in regulating gene transcription. A single bout of endurance exercise will increase AMPK and transcription and/or messenger RNA content for various metabolic and stress-related genes. Typically, transcriptional activity peaks within the first few hours of recovery, returning to baseline within 24 h. These findings have led to the overall hypothesis that training adaptations in skeletal muscle may be generated by the cumulative effects of transient increases in gene transcription during recovery from repeated bouts of exercise [17]. Although it is clear that gene transcription alone is not a guarantee that protein synthesis will occur, it is a necessary step for protein synthesis to occur. Studies have also demonstrated a link between muscle glycogen and AMPK expression, as lower muscle glycogen results in greater AMPK expression [18]. It is likely that muscle glycogen directly influences AMPK because a subunit of AMPK binds to specific glycogen-binding sites, which prevents it from being phosphorylated by upstream kinases [19]. However, when glycogen is broken down, this AMPK becomes more active [19] and with low concentrations of glycogen, high AMPK activity is observed [18, 20]. Other signaling molecules such as p38 mitogen-activated protein kinase [21] and p53 [22], as well as the expression of peroxisome proliferator-activated receptor-γ coactivator 1-alpha [23] may be enhanced to a greater extent when exercise is performed under conditions of carbohydrate restriction. It has also been demonstrated in rats that peroxisome proliferator-activated receptor-gamma transcriptional activity is sensitive to the combined effect of skeletal muscle contraction and glycogen depletion [24]. Glycogen thus plays an important role in regulating gene transcription in the muscle, which can alter protein synthesis and ultimately the training adaptation. Manipulating glycogen stores may therefore be a tool to optimize training adaptation. Training low has received considerable attention in the last few years. Here, I summarize the principles of the different methods, but for a more detailed discussion the reader is referred to several excellent recent review papers [4, 6, 7, 25–29].

#### Training Twice a Day

The first study to use this principle was a study by Hansen et al. [30] who used a one-legged kicking model to compare training daily, once a day, vs. training twice a day, every other day. The second exercise bout was performed with low muscle glycogen and essentially, therefore, subjects trained 50% of the time with low muscle glycogen. This produced marked improvements in the markers of oxidative capacity [activity of the mitochondrial enzymes 3-hydroxyacyl-CoA dehydrogenase (HAD) and citrate synthase (CS)] and increased glycogen levels compared with training in a glycogen-loaded state all the time [30]. This sparked a reaction by various researchers and coaches who argued that a single-legged kicking model did not reflect a real-life situation. In addition, the study used untrained individuals, thus the real-life relevance for athletes was still unknown. Studies with the same design and performed in parallel in the UK and Australia by Hulston et al. [31] and Yeo et al. [20] investigated the effects in a more realistic athletic setting. In both studies, cyclists trained twice a day every other day or once every day. Both studies produced similar results. The first observation in both studies was that the cyclists who trained twice a day (train low) could not maintain the same intensity as the cyclists who trained once a day. Despite the fact that the former performed less work, some of the adaptations were greater. For example, Hulston et al. [31] reported that HAD and fatty acid translocase (FAT/CD36) protein content was increased more when ‘training low’ and the ability to use fat as a fuel was improved [20, 31]. Morton et al. [32] also observed beneficial adaptations (increased succinate dehydrogenase activity) when training with low muscle glycogen. However, there were no differences in performance after 3 weeks of training low compared with the control [20, 31], but perhaps the relatively short training period in these studies was insufficient to demonstrate changes in performance. It appears that training twice a day may result in adaptations that favor fat metabolism, but it is too early to definitely conclude that this training method will also result in long-term performance benefits.

#### Training Fasted

Perhaps the most common way to ‘train low’ is training in an overnight fasted state. Typically, the last meal is consumed between 8 and 10 P.M. the night before, and exercise is performed in the morning before breakfast is consumed. This situation is different from the previous methods, where muscle glycogen was reduced by prior exercise. When training fasted, muscle glycogen should be unaffected by the overnight fast, but liver glycogen will be very low [33].

Studies by Hespel and coworkers [34, 35] demonstrated that training in the fasted state may induce more profound adaptations then training in the fed state (a carbohydrate-containing breakfast and consuming carbohydrates during exercise). For example, in one study it was demonstrated that oxidative enzymes such as CS and HAD were upregulated to a greater degree (47 and 34%, respectively) when fasted was compared with fed after 6 weeks of training (4 × per week, 1–1.5 h at 75% maximal oxygen uptake) [34]. The authors concluded that training in the fasted state was more effective to increase muscle oxidative capacity than training in the fed state. They also observed that intramuscular fat utilization was increased with fasted training and noted improvements in the regulation of blood glucose levels. The mechanisms are likely to be different from training with low glycogen. Van Proeyen et al. [36] found no differences in AMPK in subjects training in the fasted vs. fed state, but did observe differences in post-exercise eukaryotic elongation factor 2 phosphorylation (elevated after carbohydrate feeding but not after fasting). De Bock et al. [35] showed that exercise in the fasted state facilitated intramuscular fat use during exercise and improved glycogen resynthesis [35]. It was also demonstrated that carbohydrate ingestion blunted uncoupling protein 3 gene expression, whereas training in the fasted state resulted in a marked increase in uncoupling protein 3 gene expression [35]. Another study by the same research group did not result in any marked improvements with training in the fasted state [37]. In this study, small changes were observed in proteins involved in the regulation of fat metabolism but this did not result in measurable changes in fat oxidation. The results of these studies are promising and there appear to be potential benefits of training in the fasted state. However, there are still a number of practical questions that need to be answered such as how many days of training per week are needed? What is the type of training (intensity and duration) that is most suitable for fasted training? How many weeks should this training be performed to see meaningful effects? In addition, studies to date have focused on metabolic adaptations and few have addressed the potential effects on exercise performance, such as whether fasted training results in performance improvements over time?

#### Training Adaptation with Low Exogenous Carbohydrate Availability

Although the benefits of carbohydrate ingestion during exercise are generally recognized [38–41], carbohydrate supplementation during exercise may not have only positive effects. The positive effects may refer to the acute situation, but it has been suggested that chronic use of carbohydrate during exercise may limit training adaptations. This idea stems from observations that muscle glycogen stores are related to the expression of genes relevant to the adaptation to training. It is generally thought that training adaptations are the result of recurrent changes in gene expression, which occur with every bout of exercise, leading to a change in phenotype such as increases in fatty acid transport and oxidation. Long-term glucose ingestion might negatively affect the expression of relevant genes. Glucose ingestion can attenuate the rise in AMPK [42], and long-term suppression of AMPK in turn could reduce the increase in CS activity [43] and reduce muscle glycogen accumulation [44], two common markers of training adaptation. Glucose ingestion will suppress lipolysis and reduce the concentration of fatty acids in the plasma, and this possibly attenuates some of the training-induced adaptations. It has been shown that glucose ingestion during exercise may suppress the expression of carnitine palmitoyl transferase mRNA, mitochondrial uncoupling protein 3, and FAT/CD36 [45]. However, in a carefully conducted study by Akerstrom et al. [46] in which a 10-week leg extension training program was followed by the subjects, glucose ingestion did not alter training adaptations related to substrate metabolism, mitochondrial enzyme activity, glycogen content, or performance. Significant increases were observed in CS and HAD activities after the 10-week training program but there was no effect of carbohydrate supplementation on these changes. It appears that the effects of glucose ingestion during exercise were distinctly different from those induced by exercising with low muscle glycogen. It is interesting to note that Morton et al. [32] observed improvements in succinate dehydrogenase activity with low-glycogen training in the presence or absence of exogenous carbohydrate feeding.

#### Low-Carbohydrate High-Fat or Ketogenic Diets

Another way to train low would be to remove carbohydrate from the diet and have a long-term, low-carbohydrate, high-fat diet. It was demonstrated in the 1920s that reducing carbohydrate intake and increasing fat intake will result in higher rates of fat oxidation [47]. However, it was also observed that subjects felt more fatigued [47] and exercise capacity was reduced with this practice [48]. Burke and colleagues [49–52] performed a series of short-term, low-carbohydrate, high-fat diet studies and one of their observations was that 5 days on a low-carbohydrate, high-fat diet already showed some adaptations to that diet that could not be reversed completely by refilling muscle glycogen stores. Enzymes involved in fat oxidation were upregulated and fat oxidation was increased [49]. In none of the studies, however, were any improved performance effects observed [50–52]. When athletes were training over a longer period of time (7 weeks) with either a high-fat (62% fat, 21% carbohydrate) or a carbohydrate-rich (20% fat, 65% carbohydrate) diet, it was observed that both groups improved with training, but training effects were more profound in the high-carbohydrate group.

There is one study that is always referred to as evidence for the benefits of a ketogenic diet. In the 1980s, a study with five subjects showed that a ketogenic diet, containing less than 20 g of carbohydrate per day, for a prolonged period of time (4 weeks) resulted in hyperketonemia and increases in fat oxidation [53]. In this study, exercise capacity was only tested at a low intensity and showed a large degree of variation both between subjects and within subjects. On average, there was no difference in exercise capacity before and after the ketogenic diet. As expected, fat oxidation was increased and some adaptations occurred in the muscle.

A study by Stellingwerff et al. [54] demonstrated that although a high-fat diet will increase fat oxidation, perhaps by increasing enzyme activity related to fat metabolism, it can reduce enzyme activities related to carbohydrate metabolism. Thus, whilst many studies observed improvements in HAD, for example, Stellingwerff et al. [54] demonstrated compromised pyruvate dehydrogenase activity. It may therefore be that fat oxidation is increased, at least partly, as a result of an inability to use carbohydrates. Because carbohydrates are important substrates for high-intensity exercise, such adaptations would be unwanted. In fact, a carefully controlled study by Burke et al. [55] demonstrated that there were no benefits of a ketogenic vs. a high-carbohydrate diet, or a mixed approach (higher or lower carbohydrates depending on training) in elite endurance athletes. In fact, performance of high-intensity exercise was not improved by 3 weeks of intensified training in the ketogenic diet group (−1.6%), while athletes consuming the other diets made substantial performance improvements (6.6% in the high-carbohydrate group and 5.5% in the mixed group).

The ketogenic diet has received considerable attention in the popular press and many claims have been made recently. However, it is important to realize that, to date, not a single study has demonstrated performance benefits of a ketogenic diet, including the early study that is often referred to [53]. Thus, at present, there are no data on ketogenic diets in athletes on which to base performance claims.

#### Carbohydrate Restriction During Recovery

Another concept is restricting carbohydrate intake in the first hours after exercise. The time course of transcriptional activation for many exercise-induced genes stretches across the first few hours of recovery and usually returns to baseline within 24 h [56]. Traditionally, it was recommended to consume carbohydrate immediately after exercise as this results in the highest rates of glycogen synthesis [56]. When studying the effects on gene expression post-exercise with or without carbohydrates, interesting observations were made by Pilegaard et al. [57]: activation of metabolic genes was augmented following 75 min of cycling exercise when carbohydrate intake was restricted for over 5 h compared with controls [57]. Cochran et al. [21] also showed that carbohydrate ingestion post-exercise, and not necessarily changes in muscle glycogen content per se, altered the metabolic response to repeated sessions of high-intensity interval exercise. Specifically, it was observed that p38 MAPK was activated more when carbohydrate intake was restricted and this has been linked to enhanced expression of peroxisome proliferator-activated receptor-γ coactivator 1 and an improved metabolic adaptation to exercise. Other studies, however, could not confirm this and found no differences between a high- and a low-carbohydrate intake during recovery [58–60]. A recent study [60] investigated the effects of post-exercise carbohydrate intake vs. carbohydrate restriction on glycogen and gene expression. The carbohydrate intake resulted in partial glycogen replenishment but gene expression was not different between the two groups. After 24 h, glycogen replenishment was similar in the two groups (a finding that seems consistent in well-trained individuals) and gene expression levels returned to baseline levels in both groups. It is not impossible that changes in gene expression levels went unnoted because the timing of the relatively small number of sampling points may not have been perfect. Thus, the effect of carbohydrate manipulation in the recovery phase is still uncertain.

#### Sleep Low

The concept of carbohydrate restriction during recovery was extended by Lane et al. [61]. They performed the first study into the concept of ‘train high-sleep low’, which refers to a hard workout in the evening resulting in lowered carbohydrate availability (muscle and liver glycogen) followed by sleep. This practice goes against the typical advice to athletes to consume carbohydrates post-exercise (and before going to sleep) to speed up recovery. Training high and sleeping low, however, resulted in a greater upregulation of several exercise responsive signaling markers with roles in lipid oxidation the following morning compared with when an evening meal was consumed [61]. In this study, ‘train high-sleep low’ did not elicit a greater upregulation of cellular markers of mitochondrial biogenesis. The study only addressed the acute changes and did not intend to study the long-term effects on metabolism or performance.

A follow-up study performed in France studied the longer term effects of the ‘train high-sleep low’ approach. Two groups of triathletes undertook the same endurance training program for 3 consecutive weeks [62]. One group (*n* = 11) followed a ‘sleep low’ strategy for manipulating carbohydrate availability (high-intensity workout with high-carbohydrate availability followed by a carbohydrate-restricted recovery plus an overnight fast; then a prolonged submaximal workout the following morning commenced with low-carbohydrate availability) in the training schedule. The control group (*n* = 10) maintained regular carbohydrate intake throughout the day and undertook each training session with normal/high carbohydrate availability. The triathletes performed a simulated triathlon race at the start and end of the intervention period. The authors found a small but significant effect on performance as 10-km running performance increased after 3 weeks in the ‘sleep-low’ group but not in the control group. Interestingly, the authors also reported improvements in supramaximal cycling time to exhaustion with sleep low but not control.

These are the only two studies on the concept of ‘train high-sleep low’ and it may be too early to draw firm conclusions. However, the studies do provide promising results. From a practical point of view, it is important to be aware of other potential side effects and unknowns of this approach: what are the effects on recovery when applied frequently, the effects on immune function, and perhaps most importantly, the effects on quantity and quality of sleep?

### Training High

Training high refers to training with high carbohydrate availability. Muscle and liver glycogen levels are high at the start of exercise and/or carbohydrates are supplemented during exercise. There are two main reasons for using this approach. First, carbohydrates have been shown to be important to maintain the quality of endurance training [31, 63] and reduce symptoms of fatigue and overreaching [64–67]. The second reason for training high is related to intestinal function. In longer events, it is clear that ingesting carbohydrates and increasing exogenous carbohydrate oxidation will result in improved endurance performance in most events [38–41, 68]. The effects of training high on intestinal function has been discussed in detail in a separate review article in this *Sports Medicine* Supplement issue [15].

It is often argued by coaches that it is essential to maintain a high quality of training to optimize long-term training adaptations and there are a few studies that support such beliefs. Simonsen et al. [67] evaluated a group of trained rowers who performed daily hard training (twice daily) for 4 weeks whilst consuming a normal carbohydrate (5 g/kg/day) or a high-carbohydrate diet (10 g/kg/day). Mean power output in 2500-m time trials increased 10.7% in the high-carbohydrate group and 1.6% in the normal carbohydrate group. We simulated a training camp scenario where athletes performed 1–2 weeks of intensified training, resulting in extreme fatigue and decreased performance by the end of the intensified training period [64, 66]. A consistent finding in these studies was that when athletes were supplemented with carbohydrates, and had a higher overall carbohydrate intake, reductions in performance were less profound and the symptoms of overreaching were reduced, despite the fact that they performed more work in training [64, 66]. Therefore, there is evidence that during extreme training with repeated high-intensity work, a higher carbohydrate approach is preferred. However, it must be noted that these studies used an extreme training volume to simulate a training camp situation and the result after 1–2 weeks was a decreased performance in all groups. Although the train-high group seemed to recover better, the effects on longer term performance or the effects of more moderate training with higher or lower carbohydrate are understudied.

### Training the Gut

In addition to the direct effects of training high on performance, there may be other benefits through reducing GI problems. Gastrointestinal problems are very common amongst endurance athletes, ranging from mild to severe. It is possible that some of these symptoms are caused by the fact that the intestine is not adapted to absorb nutrients well under stress. It is likely that these symptoms are at least partly related to the fact that blood flow to the intestine is reduced during intense and prolonged exercise and dehydration seems to exacerbate this effect. It is also known that intestinal absorption is the main barrier to delivering carbohydrate to the contracting muscle [69]. Training the gut could potentially help with the development of gut adaptations that improve the delivery of nutrients (especially carbohydrate), and reduce the prevalence or severity of GI symptoms during exercise.

As discussed in the review article in this issue of *Sports Medicine* [15], the importance of the GI tract is often underestimated by athletes. The GI tract plays a critical role in delivering carbohydrates and fluids during prolonged exercise and can therefore be a major determinant of performance. The incidence of GI problems in athletes participating in endurance events is high [70], indicating that GI function is not always optimal in those conditions. There is a substantial body of evidence that suggests that the GI system is highly adaptable [15]. Gastric emptying as well as stomach comfort can be ‘trained’, perceptions of fullness decreased [71] and some studies have suggested nutrient-specific increases in gastric emptying [72, 73]. There is also evidence that diet has an impact on the capacity of the intestine to absorb nutrients [74]. For example, a high-carbohydrate diet will increase the number of sodium glucose co-transporter transporters in the intestine as well as the activity of the transporters [75–78], allowing greater carbohydrate absorption and oxidation during exercise [74]. It is also likely that when such adaptations occur, the chances of developing GI distress are reduced. Future studies should include more human studies and focus on a number of areas including the most effective methods to induce gut adaptations and the time line of adaptations. To develop effective strategies, it is important to obtain a better understanding of the exact mechanisms underlying these adaptations. It is clear that ‘nutritional training’ can improve gastric emptying and absorption, and likely reduce the chances and/or severity of GI problems, thereby improving endurance performance as well as ensuring a better experience for the athlete. The gut is an important organ for endurance athletes and should be conditioned for the situations it will be required to function in.

### Training Race Nutrition

Training race nutrition refers to practicing your nutritional intake plan for a race in the weeks leading up to the race. There is considerable overlap between this type of training and training the gut. If regularly performed, it is likely that adaptations in absorption and gastric emptying will occur [15]. The reverse may also be true: if certain nutrients are avoided (e.g., when following a carbohydrate-restricted diet), the capacity to absorb these nutrients is also reduced, so that in competition, less carbohydrate can be oxidized and thus intake should be reduced in competition as well.

There are other aspects that could be practiced that may affect overall performance. Examples of this include a marathon runner practicing drinking from a cup whilst running at race pace or if a runner plans to run with a bottle to also run with a bottle in training. Training race nutrition refers to mimicking everything an athlete would encounter in a race. Whereas training the gut would focus on carbohydrate absorption, for example, training race nutrition includes also drinking, ingesting salt tablets, caffeine intake, and other practices that are part of an athlete’s race day nutrition plan.

### Training Dehydrated

A concept that has been considered for some time but only recently systematically investigated is whether training in a hypohydrated state can improve performance when dehydrated. Fleming and James [79] recruited ten recreationally active individuals who performed four exercise tests in an euhydrated or hypohydrated state. Euhydration and hypohydration were induced by manipulating fluid intake in 24 h pre-exercise and a 45-min steady state run. Before and after this short training period, the subjects performed a 45-min run followed by a 5-km performance task. It was observed that dehydration reduced performance by 2.4%. The main finding, however, was that training without fluids resulted in smaller reductions in performance. On average, the runners were 5.8% slower with euhydrated training and only 1.2% slower when they trained in a dehydrated state. Additionally, the rating of perceived exertion was normalized after hypohydrated training. Thus, it appears from this study that familiarization with hypohydration may have the potential to improve performance in situations where hypohydration may occur. However, at present there is only one study to report these effects and more work is needed before we can turn these findings into clear general guidelines for athletes.

### Supplements that may Enhance Chronic Adaptations

There are a number of supplements that have been claimed to enhance training adaptations. A prime example has been leucine, an amino acid that has an important function in stimulating protein synthesis. However, many other supplements have been suggested to enhance specific aspects of metabolism. Generally, supplements linked to promoting training adaptation can be divided into three main categories based on their mechanism of action (Table 2).

**Table 2:** Categories of supplements suggested to promote training adaptations based on their mechanism of action

Supplements that may allow more training to be performed	Caffeine Bicarbonate Creatine Nitrates (beetroot)
Supplements that may initiate or increase protein synthesis and/or increase myofibrillar protein synthesis	Essential amino acids Leucine Branched-chain amino acids β-Hydroxy β-methylbutyrate
Supplements with the potential to increase mitochondrial biogenesis	Epigallocatechin gallate and green tea extracts (−)Epichatechins Resveratrol Quercetin Conjugated linoleic acid

#### Supplements that Increase Quality of Training

There are a number of supplements that claim to increase the quality as well as quantity of training, and by deduction it is then suggested that performance should also be enhanced. For both caffeine and sodium bicarbonate (NaHCO_3_), there is evidence that these supplements can enhance performance during exercise, provided the exercise duration and intensity are in the range that these supplements are effective [80]. There is also some evidence that nitrates can improve exercise performance in specific conditions [81, 82]. It must be realized that for caffeine, most studies have studied exercise lasting around 1 h and for NaHCO_3_ and nitrates typically between 1 and 10 min. Creatine has been shown to increase the sprint capacity when performing repeated sprints [83]. Such supplements could potentially improve long-term training adaptations because they would allow a greater training load or higher quality of training.

It has also been suggested that long-term NaHCO_3_ ingestion could result in improved training adaptations [12]. One study used long-term NaHCO_3_ ingestion and found that the group that consumed 400 mg of NaHCO_3_/kg body mass 1.5 and 0.5 h before interval training (6–12 × 2 min at 100% maximal oxygen uptake), three times per week over 8 weeks, had greater improvements in the lactate threshold and short-term endurance performance during high-intensity exercise (time to fatigue at 100% pre-training *V*O_2_ peak intensity) compared with the placebo group that did the exact same training [84]. The findings were not reproduced in a study in well-trained rowers [85]. Although the rowers improved over a 4-week training period, the addition of long-term NaHCO_3_ supplementation during the training period did not significantly enhance performance further. More studies are needed to confirm/or contradict the early findings but also to study the effects of long-term NaHCO_3_ supplementation on water retention and resulting changes in body mass. Increases in body mass could be unwanted in a number of settings and could be counterproductive. As discussed in Sect. 4.2, carbohydrate intake can also be used to improve the total amount of work done in training. Although there is good evidence that acutely these nutritional strategies improve performance and thus could increase the quantity as well as quality of training, there is far less evidence that such strategies also result in superior adaptations long term.

#### Supplements that Increase Protein Synthesis

There is another category of supplements that claims to increase protein synthesis (which would be relevant to both strength as well as endurance sports) or more specifically myofibrillar protein, which would benefit those athletes who want to gain muscle mass and/or strength. It has become apparent that it is mainly the essential amino acids that drive the process of muscle protein synthesis [86]. However, perhaps the most important candidate is the key essential amino acid leucine, as it alone appears to be the metabolic trigger for muscle protein synthesis [87, 88]. Leucine, branch chain amino acids, or β-hydroxy β-methylbutyrate would all potentially work through the same mechanism of ‘triggering’ (activating) muscle protein synthesis. Extensive reviews have been written on this topic and the reader is referred to these excellent papers for a detailed discussion about the factors that influence protein synthetic rates [89–91].

#### Supplements with the potential to increase mitochondrial biogenesis

This category of supplements that claims to increase mitochondrial biogenesis, fat oxidation, and endurance capacity or performance is perhaps the largest group of supplements, but it is also the one with the least solid evidence. A host of ingredients have been linked to improvements in cell signaling that could then trigger mitochondrial biogenesis. These include, but are not limited to the catechins epicatechin and epigallocatechin gallate, polyphenols such as resveratrol and quercetin, caffeine (reviewed in [10, 92]), and conjugated linoleic acid [93]. Although there are some promising results, especially in animal models, translation to healthy trained athletes is often problematic. For example, while green tea extracts (containing the active component epicatechin and epigallocatechin gallate) have been shown to increase fat oxidation and performance in mice [94], in humans, these effects were not found [95] after 7–28 days of green tea extract supplementation. Two recent review articles summarized the effects of these small nutritional bioactives [10, 92] and although there are some promising findings in some areas, the evidence does not seem convincing enough to formulate practical guidelines for the use of any of these compounds.

### Supplements that may Reduce Training Adaptation

It is important to note that several studies have suggested that certain supplements and, in particular, a high intake of antioxidants could actually reduce the training adaptation to exercise [9]. Not all studies have demonstrated such effects and differences between studies may be a function of the specific antioxidants used, and the dose and timing of intake [96]. However, more work is clearly needed and it seems wise to recommend avoiding high doses of antioxidants at this point in time if the main goals are to develop long-term training adaptations.

## Conclusion

In summary, training adaptations are the result of a complex interplay between nutrition and exercise. Therefore, by manipulating nutritional intake, it is possible to promote training adaptations. Several strategies have been developed to this effect, some of which have more evidence in support than others. The most common methods are training with high-carbohydrate availability (train high) and training with low-carbohydrate availability (train low). There are many variations of each of these methods. In addition, because the gut is an important organ, methods to improve gut function (faster absorption and reduced GI distress) have also been developed. There are also certain ingredients (supplements) that may increase the effects of training. All of these methods can be captured under the umbrella of periodized nutrition. Nutritional training is another term that is sometimes used and this term can be used interchangeably.

Which of these methods should be used depends on the specific goals of the individual and there are no methods that will address all needs. Therefore, appropriate practical application lies in the optimal combination of different nutritional training methods. In the years to come, we will undoubtedly begin to obtain a better understanding of the molecular bases for training adaptations and find ways to better incorporate and integrate periodized nutrition into training methods.
